# Steroid-Dependent Nephrotic Syndrome in a Pediatric Patient With Type-1 Diabetes Mellitus

**DOI:** 10.1155/crin/5532944

**Published:** 2025-07-10

**Authors:** Nisha S. Singh, Aubree Crabb, Ikuyo Yamaguchi

**Affiliations:** ^1^Department of Pediatrics, School of Community Medicine, The University of Oklahoma-Tulsa, Tulsa, Oklahoma, USA; ^2^Department of Pediatrics, Division of Pediatric Nephrology, The University of Oklahoma Health Sciences Center and Oklahoma Children's Hospital, OU Health, Oklahoma City, Oklahoma, USA

**Keywords:** diabetes mellitus, minimal change disease, nephrotic syndrome, pediatrics, proteinuria

## Abstract

Proteinuria in a patient with long-standing Type 1 diabetes mellitus (T1DM) usually suggests diabetic kidney disease (DKD). However, DKD occurs late in the disease and is associated with hypertension and retinopathy. We report an adolescent with T1DM who, 1 year after initial diagnosis, developed nephrotic syndrome (NS). He was treated with steroids but developed frequent relapses and became steroid-dependent. A subsequent kidney biopsy revealed minimal change disease (MCD) and mild DKD. He was treated with mycophenolate mofetil (MMF) and remains in remission. Primary podocytopathy, such as MCD, is a rare cause of NS in a patient with T1DM. Indications for kidney biopsy and treatment options are similar to those of other children with a diagnosis of NS. This report highlights that, although rare, primary glomerulopathy can occur in pediatric diabetic patients and should be considered in the differential diagnosis of proteinuria, as early recognition and intervention can lead to favorable outcomes.

## 1. Introduction

The incidence of DM is increasing in children and adolescents, with 1.9% per year for Type 1 diabetes mellitus (T1DM) and 4.8% per year for Type 2 DM (T2DM) from 2002 to 2015 [1]. Consequently, the prevalence of DKD in children is also increasing, from 1.16% to 3.44% between 2002 and 2013 [2]. DKD is usually a late complication of long-standing DM, characterized by proteinuria, hypertension, and progressive loss of kidney function. DKD is characterized by histological lesions of mesangial expansion, basement membrane thickening, and nodular lesions [3]. The average incidence of nephrotic syndrome (NS) is 2–16.9 per 100,000 children globally [4], and 90% of steroid-responsive NS is due to minimal change disease (MCD). At least 50% of children with NS will develop multiple relapses and may develop steroid-dependent NS (SDNS), defined as two consecutive relapses during steroid tapering or within 14 days of cessation of therapy [5]. Due to the increasing prevalence of DKD in children and adolescents [2], pediatric nephrologists are likely to encounter DKD with increasing frequency, and thus, it is crucial to differentiate between DKD and primary glomerulopathy, which may coexist with DM, causing proteinuria.

We present a rare case of a child with Type 1 diabetes and SDNS due to MCD and provide a review of the current literature.

## 2. Case Description

**Figure 1:**
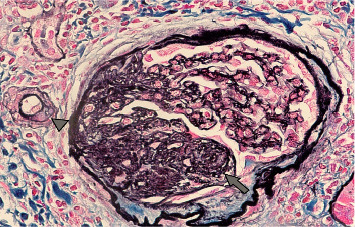
Segmental nodular mesangial expansion and sclerosis (arrow) were observed. Arterioles showed focal hilar hyalinosis (arrowhead), a feature usually seen in diabetic nephropathy.

**Figure 2:**
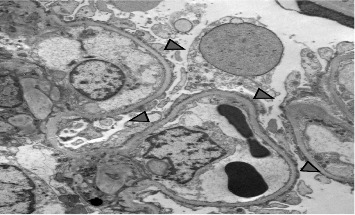
Electron microscopy revealed near-total effacement of the podocyte epithelial foot processes (arrowheads), with microvillous transformation consistent with minimal change disease.

A 15-year-old male patient was diagnosed with T1DM at 11 years of age. A year later, he developed nephrotic-range proteinuria and edema. He was diagnosed with idiopathic NS by a previous nephrologist and started on oral steroids. He developed frequent relapses and subsequently became steroid-dependent and started on mycophenolate mofetil (MMF). After they relocated, the family established care at our facility when he was 15. At presentation, he was noted to be hypertensive, with Stage-1 hypertension (131/77 mmHg); no edema was present on physical examination. Apart from insulin and MMF (600 mg/m^2^BSA/dose twice daily), the patient was not receiving any other medications. He had a history of poorly controlled DM with hemoglobin A1c (HgbA1c) in the range of 10%–11%, including admission for diabetic ketoacidosis. Pertinent labs showed low albumin (2.3 mg/dL), serum creatinine (0.5 mg/dL), urine analysis 3+ protein, no RBC, and urine protein/creatinine ratio (UPCR) of 4 mg/mg. C3 and C4 levels, hepatitis panel, and HIV testing were normal. He underwent a kidney biopsy due to recurrent relapses despite treatment with MMF. Light microscopy (Figure 1) revealed mild diabetic kidney disease with segmental nodular mesangial expansion, sclerosis, and focal hilar hyalinosis in arterioles. No Kimmelstiel–Wilson lesions were observed, and the immunofluorescence study was negative. Electron microscopy (Figure 2) revealed near-total effacement of the podocyte epithelial foot processes, with microvillous transformation consistent with MCD, normal glomerular basement membrane thickness, and the overall biopsy result was suggestive of primary podocytopathy with mild diabetic kidney disease. He was treated with oral steroids for 12 weeks with a daily taper plan, lisinopril for antiproteinuric effects and hypertension, and continued MMF. He has remained in remission (UPCR is 0.3 mg/mg) on the current therapy. His insulin therapy was increased while on steroids due to hyperglycemia, with HgbA1c between 8% and 9.7%.

## 3. Discussion

DKD is uncommon during childhood, but in susceptible patients with T1DM, DKD can begin early and accelerate during adolescence, particularly if glycemic control is poor [6–8].

Microalbuminuria is the most common finding in children, usually developing 7–10 years after diagnosis, and found in about a third of patients [3]. Overt proteinuria is found in less than 1%–1.5% of patients and often develops 10–15 years after the onset of T1DM [9, 10]. However, there are reports of young children with T1DM of short duration developing clinical DKD (within 4 years of diagnosis of T1DM) [11]. Presentation of nephrotic-range proteinuria in a patient with T1DM within a short period after diagnosis should raise suspicion of nondiabetic kidney disease and prompt consideration of kidney biopsy for diagnosis and management.

Previous reports of NS associated with T1DM have been listed in Table 1 [7, 12–20]. Among the patients reported, approximately 10 had simultaneous onset of T1DM and NS, while in nine patients, NS developed after the onset of DM. In one patient, NS developed first, followed by DM (Table 1). Although the exact association between T1DM and NS remains unclear, emerging evidence suggests a potential immunological link between the two conditions. Peces et al. reported the presence of human leukocyte antigen (HLA) DR4 and DR7 in a patient with NS and DM. Both HLA loci are known to be associated with T1DM and MCD, suggesting a genetic predisposition for the onset of NS in some patients with DM [19]. A similar report documented positivity for HLA A24, DR4, and DR53 antigens in a 3-year-old with simultaneous onset of NS and T1DM [21]. Another report described the presence of DR4 in a 4-year-old child with T1DM and NS [18].

Although MCD has been the commonest reported primary glomerulopathy in diabetic children, a recent case series of kidney biopsies in diabetic children has revealed a spectrum of glomerular pathologies. Among 17 biopsied children, four had isolated DKD, three had a combination of DKD and IgA nephropathy, and the remaining 10 were diagnosed with conditions such as MCD, membranous nephropathy, thin glomerular basement membrane disease, acute pauci-immune necrotizing crescentic glomerulonephritis, and isolated IgA nephropathy [22]. In light of such findings, the American Diabetes Association (ADA) underscores the importance of evaluating for nondiabetic kidney disease in children presenting with atypical features—such as acute kidney injury, hematuria, chronic kidney dysfunction with minimal or absent proteinuria, or the onset of NS shortly after the diagnosis of diabetes [23].

The management of NS in children with T1DM is similar to the International Study of Kidney Disease in Children (ISKDC) [5] guidelines for patients with NS. In patients with idiopathic NS, a trial of steroids is recommended, as 78.1% of children with primary NS respond to steroid therapy. The recommended prednisone dose is 2 mg/kg/day with a maximum daily dose of 60 mg/day, followed by alternate-day steroids of 1.5 mg/kg/day for another 6 weeks. High-dose steroid therapy may cause uncontrolled blood glucose levels, leading to patients requiring higher doses of insulin, as reported by Robinson and Mcconnell [12]. One patient needed to be changed from steroids to cyclophosphamide due to difficulty in controlling diabetes, as reported by Wass et al. [16]. Goldmann et al., in their series, experienced difficulty controlling blood glucose and suggested using daily steroid tapering instead of alternate-day steroid tapering [13]. While these patients are on steroids, working closely with endocrinologists to optimize blood glucose control is crucial.

The Glassock et al. working group recommended kidney biopsy in patients with T1DM if a patient with a disease duration of less than 10 years develops clinical evidence of kidney disease regardless of age, sex, race, or ethnicity [24]. Goldmann et al. opined that the same statement may not be appropriate for children with T1DM and SSNS [13]. Biopsy results usually have no influence on treatment decisions in patients with SSNS and are recommended in children who are steroid-dependent/frequent relapsers or have steroid-resistant NS (SRNS) [5]. In previous case reports, four patients had SDNS, and kidney biopsy was performed in two of those patients, both of whom had MCD and were managed with steroid-sparing agents such as cyclophosphamide, cyclosporine, or levamisole. Our patient also had MCD histology along with early diabetic kidney disease changes. He was started on MMF as a steroid-sparing agent and has been in remission for at least 12 months with occasional relapse, which was treated with a short course of steroids with tapering.

Kari et al. reported two patients with simultaneous onset of SRNS and T1DM, with histology consistent with MCD [17]. Genetic testing for NPHS2 and WT1 gene mutation was negative. Proteinuria was well controlled at 1-year follow-up with cyclophosphamide, cyclosporine, and angiotensin-converting enzyme (ACE) therapy. A follow-up biopsy after 5 years in one of the patients showed findings consistent with DN.

In summary, early-onset nephrotic-range proteinuria in pediatric patients with T1DM should prompt investigation for nondiabetic kidney disease such as MCD. It is crucial to differentiate between DKD and primary glomerulopathy in T1DM, as treatment with immunosuppressants can be an option for children with primary glomerulopathy with a better prognosis, compared to supportive care for DKD. Management of NS and indications for kidney biopsy in nephrotic children with and without T1DM are similar [13]. Steroid-sparing agents such as MMF and/or rituximab may be considered for patients with SDNS or frequently relapsing NS. Close attention should be paid to glycemic control while on high-dose steroids.

## Figures and Tables

**Table 1:** Review of case reports of children with nephrotic syndrome associated with Type 1 diabetes mellitus in pediatric patients.

Authors	DM onset (years)	NS onset (years)	Renal biopsy	Treatment	Outcome
Urizar et al. (1969)	3.3	4.3	MCH	Steroid	Resolved
	4.5	4.5	MCH	Insulin	Resolved
	5	5	MCH	Steroid	Recurrence
	8	8	MCH	Steroid	Resolved
	0.16	10	MCH + DKD	Steroid	Recurrence

Robinson et al. (1997)	3	3	Immune complex GN, mesangial pattern	Steroid	Resolved

Wass et al. (1978)	13.2	14	MCH	Steroids cyclophosphamide	Resolved

Peces et al. (1987)	9	3	MCH	SDNS cyclophosphamide	Relapsed

Gilboa et al. (1979)	3	7	MCH + DKD	Steroids and chlorambucil	Resolved

Robinson and McConnell (1961)	8	8	NA	Steroids	Relapsed

Goldman et al. (2001)	3.3	3.8	MCH	SDNS-cyclosporine	Relapsed
	9	15.5	MCH + early DKD	Steroids	Relapsed
	0.11	8	NA	Steroids	Resolved (8)
	4.3	4.3	NA	SDNS-levamisole	

Agres et al. (2006)	3	3	NA	Steroid	Relapsed

Rego Filho et al. (2003)	3	3		Steroid cyclophosphamide	Relapsed

Moyses Neto et al. (2012)	15	19		Steroid	Resolved

Bawahab et al. (2019)	9	12	NA	Steroids	Resolved

Kari et al. (2010)	3	3	MCH	SRNS, cyclophosphamide	Remission
	1.8	1.8	MCH	SRNS cyclosporine, ACE	Remission

Abbreviations: ACE, angiotensin-converting enzyme inhibitor; DKD, diabetic kidney disease; DM, diabetes mellitus; GN, glomerulonephritis; MCH, minimal change histology; NA, not applicable; NS, nephrotic syndrome; SDNS, steroid-dependent nephrotic syndrome; SRNS, steroid-resistant nephrotic syndrome.

## Data Availability

The data that support the findings of this study are available on request from the corresponding author. The data are not publicly available due to privacy or ethical restrictions.
